# Plant Growth-Promoting Bacteria: Biological Tools for the Mitigation of Salinity Stress in Plants

**DOI:** 10.3389/fmicb.2020.01216

**Published:** 2020-07-07

**Authors:** Akhilesh Kumar, Saurabh Singh, Anand Kumar Gaurav, Sudhakar Srivastava, Jay Prakash Verma

**Affiliations:** Institute of Environment and Sustainable Development, Banaras Hindu University, Varanasi, India

**Keywords:** microbiome, plant growth-promoting bacteria, salinity stress, salt stress amelioration, sustainable agriculture

## Abstract

Salinity stress is one of the major abiotic stresses threatening sustainable crop production worldwide. The extent of salinity affected area is expected to cover about 50% of total agricultural land by 2050. Salinity stress produces various detrimental effects on plants’ physiological, biochemical, and molecular features and reduces productivity. The poor plant growth under salinity stress is due to reduced nutrient mobilization, hormonal imbalance, and formation of reactive oxygen species (ROS), ionic toxicity, and osmotic stress. Additionally, salinity also modulates physicochemical properties and reduces the microbial diversity of soil and thus decreases soil health. On the other hand, the demand for crop production is expected to increase in coming decades owing to the increasing global population. Conventional agricultural practices and improved salt-tolerant crop varieties will not be sufficient to achieve the yields desired in the near future. Plants harbor diverse microbes in their rhizosphere, and these have the potential to cope with the salinity stress. These salinity-tolerant plant growth-promoting bacteria (PGPB) assist the plants in withstanding saline conditions. These plant-associated microbes produce different compounds such as 1-aminocyclopropane-1-carboxylate (ACC) deaminase, indole-3-acetic acid (IAA), antioxidants, extracellular polymeric substance (EPS), and volatile organic compounds (VOC). Additionally, the naturally associated microbiome of plants has the potential to protect the host through stress avoidance, tolerance, and resistance strategies. Recent developments in microbiome research have shown ways in which novel microbe-assisted technologies can enhance plant salt tolerance and enable higher crop production under saline conditions. This focused review article presents the global scenario of salinity stress and discusses research highlights regarding PGPB and the microbiome as a biological tool for mitigation of salinity stress in plants.

## Introduction

**FIGURE 1:**
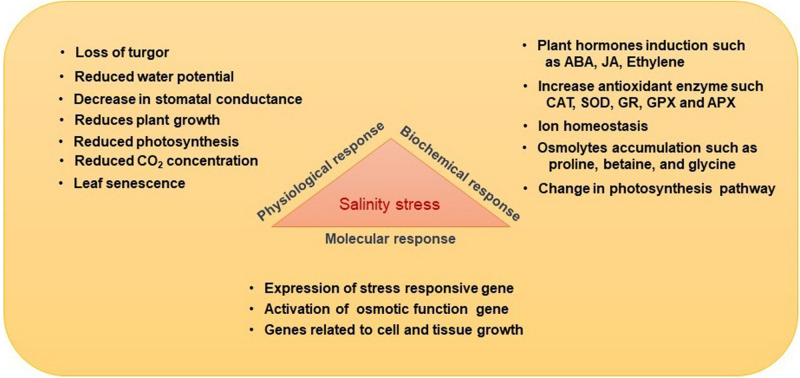
Effects of salinity stress on different plant attributes.

**FIGURE 2:**
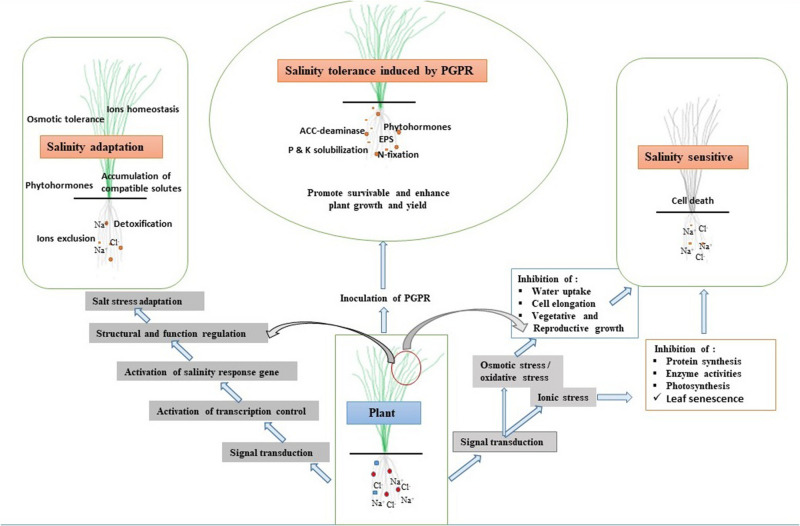
Effects of salinity stress on plant signaling mechanism, adaptation, and PGPR-induced stress tolerance.

A major challenge for world agriculture is to fulfill the food demand of the increasing global population, which is currently growing at a rate of around 1.05% per year (World Population Prospects, 2019). Plant growth, productivity, yield, and food quality are severely influenced by several biotic and abiotic stresses (Shi-Ying et al., 2018; Singh et al., 2018). The biotic stresses include damages or infections caused by various pests or pathogens. The abiotic stresses include drought, salinity, temperature, heavy metals, and other organic contaminants. Among all abiotic stresses, soil salinization is the most detrimental (Daliakopoulos et al., 2016) and is considered to be one of the most significant limiting factors of agricultural productivity and food security. Worldwide, about 20% of agricultural land is affected by salinity, and this is continuously increasing (Gupta and Huang, 2014). It is estimated that by 2050, about 50% of agricultural land will be salinity affected. Salinization of agricultural land occurs mostly due to the accumulation of salts in soil (Bharti et al., 2016; Shi-Ying et al., 2018), particularly sodium (Na^+^) and chloride (Cl^–^) ions. High Na^+^ accumulation limits water conductance, soil porosity, and aeration. In addition, soil salinity stress negatively affects the microbial diversity within and around the roots of plants. A plant under salinity stress undergoes several morphological, physiological, and molecular changes, which impede its growth and development (Figure 1). Besides, a high salt concentration affects enzyme activities, stomatal conductance, and the rate of photosynthesis (Kumar and Verma, 2018). Salinity stress also causes oxidative stress by enhancing the production of reactive oxygen species (ROS), which damage cell membranes, proteins, lipids, and nucleic acids (DNA, RNA) and may also induce programmed cell death (Figure 2). Salinity also leads to hypertonic stress due to the excessive accumulation of Na^+^ and Cl^–^ ions (Shi-Ying et al., 2018).

Many salt-tolerant crops varieties have been developed through transgenic technologies and conventional breeding approaches. However, these two approaches are insufficiently, labour-intensive and time-consuming. In light of upcoming challenges, it has now become necessary to use alternative technologies simultaneously to promote sustainable agriculture, such as the use of plant growth-promoting bacteria (PGPB). The rhizosphere has complex microbial diversity, which may be considered as the natural relations between plants and microbes. Several recent studies have revealed that PGPB act as elicitors of salinity tolerance in plants and promote their growth (Vacheron et al., 2013; Daliakopoulos et al., 2016; Tiwari et al., 2016). Mostly, PGPB reside around the root zone of plants in saline soil. The various PGPB-mediated mechanisms include biofilm formation, extracellular polymeric substance (EPS) production, nitrogen fixation, phytohormone production, and ACC-deaminase activity (Ansari et al., 2019). PGPB also promote nutrient uptake and homeostasis in plants and increase antioxidant activities during salt stress. Plant growth-promoting bacterial endophytes reside in healthy plant tissues without causing them any disease. These endophytic PGPB can also promote salinity stress tolerance and plant growth (Ali et al., 2014).

Kumar et al. (2020) reported that the growth and yield of French bean (*Phaseolus vulgaris*) are optimized by the application of PGPR consortia against salt stress. Siderophore-producing rhizobacteria may represent a promising alternative to chemical fertilizers due to simultaneously tackling salt-stress effects and enhancing available iron in saline soils (Ferreira et al., 2019). In response to higher salinity stress, resistance is achieved by a change in membrane transport properties such as the regulation of Na^+^/H^+^ antiporters and various ion channels (Kumar et al., 2017). Besides, transcription factors (TFs) play a vital role in providing tolerance against salinity stress through regulation of the expression of stress-related genes. There are several TF families; among these, NAC, AP2/ERFBP, bZIP, MYB, and WRKY have been identified as potential players for improving crop tolerance against salinity stress (Barnawal et al., 2017; Tang et al., 2019). Further, PGPB also control plant pathogens through the production of antibiotics and competition for the ecological niche and nutrients (Kumar and Verma, 2018). Therefore, the interaction between plant and PGPB is a part of adaptation due to the mutagen effect (Nautiyal et al., 2008; Shi-Ying et al., 2018). Thus, to provide a sustainable solution for agriculture and cope up with salinity stress, it is necessary to explore the diversity of the microbes so as to understanding their physiological and functional features and harness their potential.

### Salinity Status in India and the World

**FIGURE 3:**
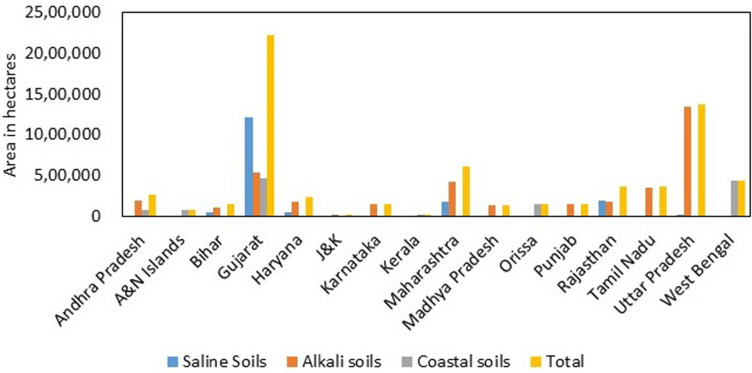
Extent and distribution of salt-affected soils in India (Central Soil Salinity Research Institute [CSSRI], 2019).

**FIGURE 4:**
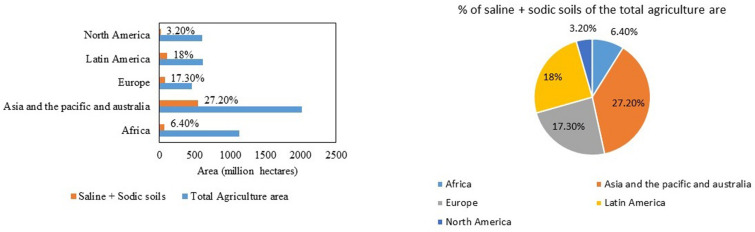
Area of the total agricultural land in different continents that has sodic and saline soil (in millions of hectares of land) (FAO, 2019; Chauhan et al., 2019).

India has a mainland coastline of 6100 km, which is prone to salinity problems. Apart from that, regions away from the coast also experience salinity issues. In India, about 6.7 million ha of land are affected by salinity (Narayana and Babu, 1983). The affected soils are divided into three major categories: saline soils, alkali soils, and coastal soils. Gujarat has the highest amount (almost 71% of the total saline soils in India) of salt-affected soils (1.2 million ha). The states affected with saline soils are in the following order, Gujarat > Rajasthan > Maharashtra > Haryana > Bihar > Uttar Pradesh > Karnataka (Figure 3). The problem of alkali soils is mainly faced by Uttar Pradesh, which accounts for almost 36% of the total alkali soils in India. The states falling next in the list are Gujarat, Maharashtra, Tamil Nadu, Andhra Pradesh, Haryana, Rajasthan, Punjab, Karnataka, Madhya Pradesh, Bihar, and Jammu and Kashmir. Gujarat also has the maximum (0.5 million ha) coastal salt-affected soil in India: about 37% of the total coastal salt-affected soils of India. Internationally, the most salinity-affected regions include the Asia Pacific and Australia. These two continents cover a total agricultural area of 2016.63 million ha, out of which 27% (549.30 million ha) is salinity affected. In Africa, 72.2 million ha of land are salinity affected, which is approximately 6.40% of the total agricultural area. The Americas have a total of 1223.41 million ha of total agricultural area, of which 130.5 million ha of land is saline. In Europe, 17.30% of the land area faces the problem of salinity (Figure 4; FAO, 2019; World Population Prospects, 2019).

### Effects and Causes of Soil Salinity

The world is saline; this is evident from the fact that, on average, the oceanic waters have a salinity of about 3.5%, which constitutes about 96.5% (roughly 1.3 billion km^3^) of the Earth’s water (Kırtel et al., 2018). Apart from this, roughly 12.87 million km^3^ of saline water is groundwater, and 85400 km^3^ of saline water is found in lakes (Eakins and Sharman, 2010; Kırtel et al., 2018). The acceptable limit of electrical conductivity (EC) causing no damage to crops is <0.7 dS/m, while EC over 3.0 dS/m severely affects the crop yield (Ayers and Westcot, 1985). The EC of seawaters is approximately 10–100 dS/m (Wang et al., 2006). The accumulation of salts in water affects the ability of plants to extract water from the soil. The TDS value considered suitable for irrigation water is <450 mg/l (Ayers and Westcot, 1985), while its value in seawater ranges between 5000 and 70000 mg/l (0.5 and 7%) (Abdel-Aal et al., 2010). Thus, there is a huge problem with saline soil conditions in areas near coasts, which are mostly irrigated with mixed waters from the sea or oceans that have a high concentration of salts.

The salinity of soil can be broadly classified into two categories, natural and anthropogenic. Natural processes such as rainfall and its subsequent evaporation and derivation of the soil from a saline parent material are the most important factors. Some soils also contain natural salt deposits, which lead to saline conditions in the soil. Many times, soils in coastal areas are saline because of the sea sprays received. The salts from salt-affected land can also seep into lowland areas nearby through irrigation water. Ancient natural fossil salt deposits are found in arid and semi-arid regions. Saline material is sometimes found beneath the top layer of soil, which creates a highly concentrated solution when it is dissolved in water. When this reaches the topsoil, either by groundwater pumping or surface streams in lowland areas, it creates saline soil conditions. Water evaporation takes place in the pure state, which leaves behind salts in the soil. As a result of irrigation with salty water and subsequent evapo-transpiration, the salt concentration in soil continues to increase (Carter, 1975). Groundwater irrigation also causes saline conditions when it contains high salt concentrations from natural deposits of high-salt minerals.

In water stress conditions, phreatophytes growing along canals elevate the levels of salts by consuming water and leaving the salts behind in the remaining water used for irrigation. Such plants are found along the canals and drains of irrigated areas (Carter, 1975). The geographical location of a place also plays a role in its salinization. The downstream region of the Indo-Gangetic plain, especially the Bengal flood plain, suffers mainly because of its geographical location. The sedimentation from the Ganga and the Brahmaputra rivers is another way through which saline conditions are created in some parts of Bangladesh (Mahmuduzzaman et al., 2014). A high level of sedimentation in the region causes waterlogging and flooding with saline water due to blocked rivers and upstream drainage congestion.

### Impacts of Salinity on Soil and Plant

Surface crusting is one of the impacts of salinity on the soil. Crusting is the process of formation of hard white layers over the surface of the soil in the early growing seasons, especially when the soil is without the canopy cover of the crops. Surface crusting is driven by many factors. Based on the dispersion method, it is categorized into physical and chemical dispersion. Physical dispersion is caused by the impact of raindrops and irrigation water, which destroys weak soil aggregates, creating a surface seal, also known as crusting (Warrence et al., 2002). Chemical dispersion is caused by the water due to its ESP and EC. Soil crusts consist of two distinct parts, an upper skin (0.1 mm thick) caused by the impact of raindrops and a washed-in layer caused by the accumulation of clay particles. It is found that soils that disperse easily are 1000 times more permeable than the average crust. The surface crust effectively seals off the subsoil hydraulically, creating more runoff potential on the upper crust (Hardy et al., 1983). Farmers quite often use rotary hoes to till the surface crust, which is a short-term solution. Residue cover acts as a shield to prevent the direct impact of raindrops on the soil surface and provides important pathways for water entry into it. Soil salinity reduces the content of nutrients and microbial diversity in the salinity-affected area. The organic matter, nitrogen, dissolved organic carbon, and microbial carbon biomass (MCB) of soil are highly affected by salinity (Xu et al., 2020). Further, microbial activities such as soil respiration and soil enzyme activity are depressed by salinity. Thus, salinization of soil is recognized as a major threat to agricultural activity, human resources, and health (Shrivastava and Kumar, 2015).

Salinity affects almost every aspect of plant morphology, physiology, and biochemistry and thus causes significant loss of crop yield. A higher concentration of salt in soil restricts the uptake of water and essential nutrients by plant roots. The higher concentration of ions (Na^+^) in root causes osmotic stress, decreases water potential, and disturbs the nutrient balance. Also, a higher Na^+^ concentration outside the plant cell negatively impacts intracellular K^+^ influx, which is an essential element required for plant growth. Moreover, excess Na^+^ concentration inside the cell causes various physiological disorders, such as reduced seed germination, seedling growth, flowering, and fruiting (Singh et al., 2015). Excess salt also decreases the pigment (chlorophyll) content of the leaves, the leaf area, and photosynthetic efficiency. The inhibition of photosystem II (PSII) activity, which is a major site of the electron transport chain (ETC), occurs during salinity stress (Mehta et al., 2010; Kalaji et al., 2011). Several cellular enzymes are affected by salinity stress, such as RNase, DNase, proteases, and enzymes involved in nitrogen metabolism and synthesis of amino acids (Nathawat et al., 2005; Siddiqui et al., 2008). Salinity stress also indirectly induces the accumulation of ROS, such as singlet oxygen, superoxide radicals, and H_2_O_2_. The ETC in the chloroplast and mitochondria are major sites of ROS production under salinity stress conditions (Gill and Tuteja, 2010).

## Signaling Mechanisms

**FIGURE 5:**
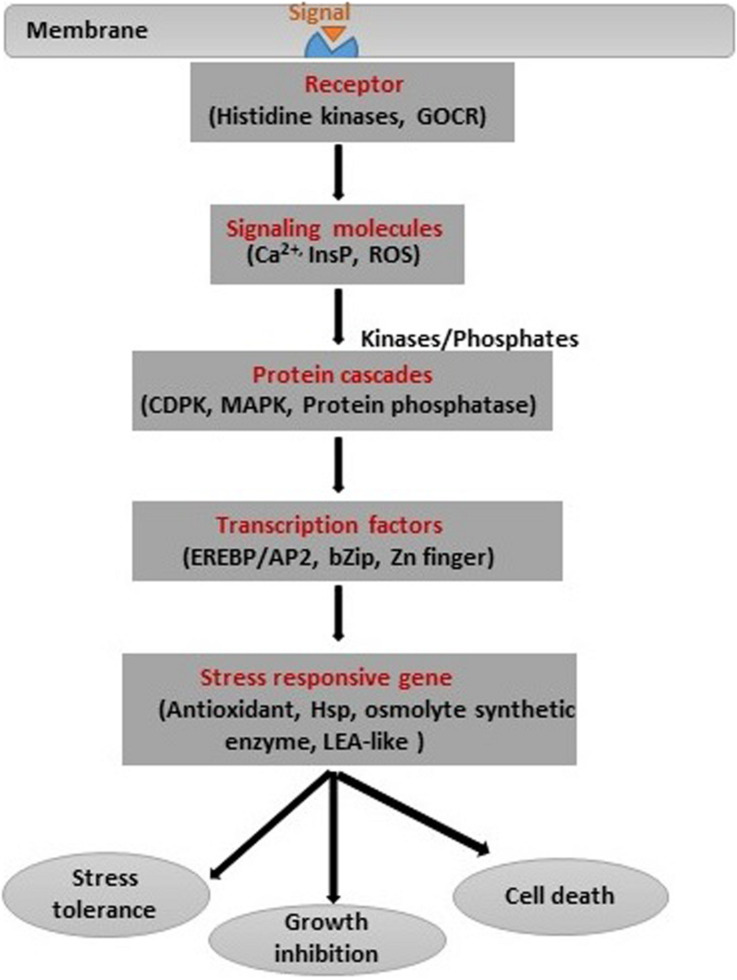
Stress signal transduction and different signaling intermediates.

Plants adopt various mechanisms to survive under salinity stress through modifications at the morphological, physiological, and biochemical levels. These diverse modifications require modulation in different stress-related genes involved in regulatory and signaling pathways (Bharti et al., 2016; Numan et al., 2018). It is fundamental to understand the mechanisms involved in signaling of salinity stress in plants as well as between plant and bacteria (Figure 5). In general, plant signal transduction starts with the perception of signals by receptors on the surface, and this is followed by the generation of secondary messengers like inositol phosphates and ROS (Deinlein et al., 2014). The secondary messengers target proteins like CDPK (calcium-dependent protein kinases), MAPK (microtubule-associated protein kinase), and protein phosphatase involved in the control of gene expression through modulation of Ca^+^ concentration. There are other genes involved in the regulation of plant hormones and other cellular activities (Miller et al., 2010). Transcription factors play a unique role in salinity stress tolerance in crops because of their unique roles in modulating different stress-responsive genes. These TFs include a large number of families, like AP2/ERF, bZIP, MYB, NAC, and WRKY, which modulate the expression and function of many genes. The modulation of the salinity stress-related genes also depends on the post-transcriptional modulation of TFs. The role of TFs in the expression of the various genes and salinity stress tolerance has been extensively studied. The overexpression of *bZIP* gene in *Tamarix hispida* (Wang et al., 2010) and *CkdREB* gene in *Caragana korshinskii* (Wang et al., 2011) gave resistance against salinity stress. PGPB and endophytes also play a significant role in inducing plant signaling under salinity stress conditions. For instance, PGPR such as *Arthrobacter protophormiae* (SA3) and *Dietzia natronolimnaea* (STR1) enhanced salinity stress tolerance in wheat plants by modulating the expression of a regulatory component CTR1 (Constitutive Triple Response1) of the ethylene signaling pathway and DREB2 TF (Barnawal et al., 2017). Kim et al. (2014) found that *Enterobacter* spp. Increased the expression of salt stress-responsive genes such as *DREB2b, RD29A, RD29B*, and *RAB*18 in *Arabidopsis* under salinity stress. In another study, *D. natronolimnaea* was found to induce the expression of *TaMYB* and *TaWRKY* genes in wheat (Bharti et al., 2016). Further, genes related to antioxidants, HSP, and osmolyte synthetic enzymes are activated in response to salinity stress. However, salinity stress signal transduction has remained intriguing owing to its complexity and commonality with drought stress signals (Joshi et al., 2019).

In addition, epigenetic processes such as DNA methylation and post-translational modifications of histones influence the efficiency of stress-induced gene expression under salinity stress (Dietz et al., 2010; Golldack et al., 2011). However, the expression of salinity-related genes and protein translation depends on the type of plant. For instance, expression of TF *bZIP24* induced transcription in the salt-sensitive species of *Arabidopsis* but repressed it in salt-tolerant species (Miller et al., 2010; Golldack et al., 2011). The emerging tools, such as genomics, transcriptomics, and proteomics, will help in understanding plant stress signaling in response to salinity in greater detail in the future.

## Microbial Resistance and Resilience Under Salinity Stress

There is increasing interest in understanding modifications to rhizospheric microbial diversity and community structure in response to different abiotic and biotic stresses. The ability of diverse soil microbial communities to withstand changes in environmental conditions can be described in terms of resistance and resilience. The term resistance describes the ability of a microbial community to avoid changes in its community structure in the presence of an environmental stressor, while resilience describes the ability of the microbial community to return to its original state when stress is absent and original environmental conditions are re-established (Allison and Martiny, 2008). Salinity stress affects microbial properties, community structure, and functions. However, various processes can continue at the same rate if the community contains a high degree of functional redundancy. One microbial taxon can be replaced by another with the potential to tolerate and survive under the stress; the new bacterium can then continue to perform the same functions. The salinity-resistant microbial community improves the health of salinity-impacted soil, maintains ecological functions, and sustains and promotes the growth of plants (Mbodj et al., 2018; Kumar and Verma, 2019).

Salt-tolerant bacteria can survive in different salt concentrations (30%) and overcome the effect of salt by different mechanisms, such as accumulation of compatible solutes for osmoregulation, production of extracellular proteases, and activation of Na^+^/H^+^ antiporters. *Staphylococcus epidermidis* (P-30) can survive in high salt concentration (up to 20%) and possesses plant growth-promoting properties (Das et al., 2015). The use of such salt-tolerant bacteria for inoculation or the use of their genes for the development of transgenic plants have been found to be successful in imparting salt stress tolerance in plants. For instance, the *codA* gene of *Arthrobacter globiformis* encoding choline oxidase was expressed in tomato (*Lycopersicon esculentum*). It induced the synthesis of glycine betaine and improved the salinity tolerance of the plants (Goel et al., 2011). In another study, it was found that inoculation of *D. natronolimnaea* STR modulated the transcriptional machinery responsible for salinity tolerance in wheat plants, such as salt overly sensitive (SOS) pathway-related genes (SOS1 and SOS4). Besides, enhanced gene expression of various antioxidant enzymes such as ascorbate peroxidase (APX), catalase (CAT), and superoxide dismutase (SOD) and higher proline content in PGPR-inoculated wheat plants were also observed (Bharti et al., 2016). Hence, the application of beneficial stress-tolerant microbes not only helps in improving the microbial community structure but also in enhancing plant and soil health under salinity. Further research will be required to reveal the hidden mechanisms of stress-tolerant microbial diversity.

## PGPB as a Salinity-Alleviating Agent

### Phytohormone Production and ACC-Deaminase Activity

Plant growth-promoting bacteria are known to increase the growth of the plant by the production of hormones such as auxin, cytokinin, and gibberellin and the reduction of ethylene by ACC deaminase. Ethylene is a gaseous hormone that is known to accumulate in plants under abiotic stress conditions. The level of ethylene accumulation in plants varies in different species, genus, organs, and tissues. Ethylene is involved in growth and developmental processes, such as seed germination, root hair development and elongation, fruit ripening, leaf abscission, and organ senescence (Ahmad et al., 2011), through the regulation of several stress-related genes. Therefore, ethylene is essential for plant growth and development. However, the higher concentrations of ethylene accumulation under stress conditions can become detrimental and inhibit plant growth (Erice et al., 2017). PGPB regulate the ethylene level in plants through ACC deaminase, which cleaves ethylene precursor ACC to ammonia and α-ketobutyrate and consequently facilitate plant growth and confer stress tolerance (Table 1; Ansari et al., 2019). PGPB with ACC deaminase activity modify the number of root tips and their surface area. Hence, PGPB promote nutrient acquisition and survival under stress conditions. It is reported that the production of ACC deaminase enzyme and a decrease in the level of ethylene are the main reasons for PGPB-mediated plant growth promotion under salinity stress (Bhise et al., 2017). For example, *Pseudomonas syringae* in moong (Ahmad et al., 2011), *Rhizobium phaseoli* in bean (Ahmad et al., 2011), *Pseudomonas fluorescens* in groundnut (Saravanakumar and Samiyappan, 2007), and *Pseudomonas putida* in canola (Cheng et al., 2007) effectively alleviated salinity stress (Table 1).

**TABLE 1:** Mechanisms of salinity stress tolerance in different plants.

**Crops**	**Bacterial Strains**	**Mechanism/Action**	**References**
**Cereals**			
Maize (*Zea mays* L.)	*Rhizobium* and *Pseudomonas*	Increase proline synthesis, maintain the water level and selective uptake of ions, and decrease electrolyte leakage	Bano and Fatima (2009)
	*Bacillus* sp. and *Arthrobacter pascens*	Promote plant growth by phosphate solubilization and siderophore production under salt stress	Ullah and Bano (2015)
Rice (*Oryza sativa* L.)	*Bacillus amyloliquefaciens*-SN13	During salinity stress, increase plant biomass, water content, and proline and decrease reactive oxygen activities	Chauhan et al. (2019)
	*Bacillus amyloliquefaciens NBRISN13*	Modify gene expression and microbial community in the rhizosphere	Nautiyal et al. (2013)
	*Pseudomonas alcaligenes* and *P*. *pseudoalcaligenes*	Alleviate the harmful effects of salinity stress and maintain bacterial diversity in the rhizosphere	Rangarajan et al. (2002)
	*Pseudomonas pseudoalcaligenes* and *Bacillus pumilus*	Reduce lipid peroxidation and superoxide dismutase activity and promote plant growth and development	Jha and Subramanian (2014)
Barley (**Hordeum vulgare** L.)	*Hartmannibacter diazotrophicus*	Ameliorate salt stress through ACC deaminase activity	Suarez et al. (2015)
Wheat (**Triticum aestivum** L.)	*Pseudomonas pseudoalcaligenes*	A higher concentration of glycine betaine-like quaternary compounds and higher shoot biomass at lower salinity levels	Jha et al. (2011)
	*Bacillus amyloliquefaciens* NBRISN13	Modulates the gene expression profile of leaf and rhizosphere community	Nautiyal et al. (2013)
	**Azotobacter vinellandii** (SRI**Az**3)	Higher IAA, gibberellins (GA_3_), zeatin (Zt), proline, and malondialdehyde	Sahoo et al. (2014)
	**Bacillus pumilus**	Limits uptake of toxic ions and increases production of antioxidants	Khan et al. (2016)
	*Burkholderia* sp. MTCC 12259	Enhances production of ACC deaminase	Sarkar et al. (2018)
	*Curtobacterium albidum* strain SRV4	Increases nitrogen fixation, exopolysaccharide (EPS), hydrogen cyanide (HCN), and IAA production, and ACC deaminase activity under salinity stress	Vimal et al. (2019)
	*Sphingomonas pokkalii* sp.	Modulates rice gene and regulates the negative effect of salinity stress	Palaniyandi et al. (2014)
	*Pseudomonas pseudoalcaligenes* and *Bacillus pumilus*	Reduce lipid peroxidation and superoxide dismutase activity	Jha and Subramanian (2014)
	*Bacillus subtilis* SU47 and *Arthrobacter* sp. SU18	Increase total soluble sugars, proline content, and dry biomass	Upadhyay et al. (2012)
	*Dietzia natronolimnaea* STR1	Modulates the expression of stress-responsive genes, involving induction of *TaMYB* and *TaWRKY* expression	Bharti et al. (2016)
	**Bacillus pumilus** strain FAB10	Biofilm formation on the root surface, enhanced amount of EPS, IAA, ACC deaminase activity, and solubilized phosphate	Ansari et al. (2019)
	**Serratia** sp. SL- 12	Production of ACC deaminase and promotion of plant growth promotion under salinity stress	Singh and Jha (2016)
	*Klebsiella* sp. SBP-8	Reduces salinity effects by ACC deaminase activities and induces systemic tolerance	Singh et al. (2015)
	*Arthrobacter protophormiae* (SA3) and *Dietzia Natronolimnaea* (STR1)	Enhance photosynthesis, level of IAA, reduce abscisic acid (ABA)/ACC content, and modulate the expression of a regulatory component (*CTR1*) of the ethylene signaling pathway	Barnawal et al. (2017)
	*Chryseobacterium gleum* sp. SUK	ACC deaminase activity, production of IAA, siderophore, ammonia, and hydrogen cyanide, promoting plant growth under salinity stress	Bhise et al. (2017)
	*Azospirillum* strains	Significantly increases shoot dry weight and grain yield	Nia et al. (2012)
**Pulses**			
Mung bean (*Vigna radiata* L.)	*Pantoea* sp. and *Enterococcus*	Increase salinity tolerance due to ACC deaminase activity and plant growth promotion	Panwar et al. (2016)
	*Pseudomonas syringae*; *Pseudomonas fluorescens*; *Pseudomonas fluorescens* and *Rhizobium phaseoli*	Contain ACC deaminase, reduce ethylene production, and promote nodulation under salinity stress condition	Ahmad et al. (2011)
*Peanut (Arachis hypogaea* L.)	*Brachybacterium saurashtrense*, *Brevibacterium casei*, and *Haererohalobacter*	Halotolerant PGPR promote plant length, shoot length, root length, and total biomass under saline conditions	Shukla et al. (2012)
Soybean (*Glycine max* L.)	*Pseudomonas putida* H-2-3	Increases chlorophyll content and length and fresh and dry weight of shoots	Kang et al. (2014b)
Pea (*Pisum sativum* L.)	*Arthrobacter protophormiae*	Improves colonization of diverse bacterial population, ACC deaminase activity, and protection against salinity stress	Barnawal et al. (2014)
**Vegetables**			
Cucumber (**Cucumis sativus** L.)	*Burkholderia cepacia* SE4, *Promicromonospora* sp. SE188 and *Acinetobacter calcoaceticus* SE370	Reduce concentration of sodium ions, catalase, peroxidase, polyphenol oxidase, and total polyphenol, while potassium and phosphorus are abundantly available. Reduced level of ethylene content in plant under salt stress	Kang et al. (2014a)
Lettuce (*Lactuca sativa*)	*Azospirillum*	Increases ascorbic acid content in response to salinity stress	Fasciglione et al. (2012)
	*Pseudomonas*	Induction of antioxidant enzyme system	Kohler et al. (2009)
Tomato (*Solanum lycopersicum* L.)	*Streptomyces* sp.	Production of proline and ACC deaminase and promotion of plant growth	Palaniyandi et al. (2014)
	*Sphingomonas* sp.	Production of EPS and antioxidants	Halo et al. (2015)
	*Enterobacter* spp.	Increases IAA production, induces expression of salt stress-responsive genes such as *DREB2b, RD29A*, and *RD29B*	Kim et al. (2014)
Beet (*Beta vulgaris* L.)	*Micrococcus yunnanensis*, *Planococcus rifietoensis*, and *Variovorax paradoxus*	Growth-promotion under salinity conditions with the help of nitrogen fixation, production of IAA and siderophores, phosphate solubilization, and ACC deaminase activity	Zhou et al. (2017)
**Others**			
Cotton (*Gossypium hirsutum* L.)	*Pseudomonas*	Salinity tolerance by the modulation of phytohormone IAA	Egamberdieva et al. (2015)
Peppers (**Capsicum annuum** L.)	*Bacillus*	ACC deaminase activity promotes salinity stress tolerance and reduces ethylene in plant	Wang et al. (2018)
**Arabidopsis thaliana**	*Bacillus megaterium*	Upregulation and adjustment of jasmonic acid (JA) metabolism	Erice et al. (2017)
	**Bacillus amyloliquefaciens** FZB42	Promotes salt adaptation through regulation of transcripts associated with phytohormones, photosynthesis, osmoprotectant synthesis, and translocation of Na^+^ ions	Liu et al. (2017)
	**Klebsiella** sp.	Modulates *rbcL* and *WRKY1* genes expression	Sapre et al. (2018)
	*Hartmannibacter diazotrophicus* E19	Production of EPS and ACC deaminase	Suarez et al. (2015)

Auxins are the other major plant hormones that are regulated by PGPB. Auxins are the group of hormones like indole-3-butyric acid (I3B) and indole-3-acetic acid (IAA). Bacteria producing IAA include *Actinobacteria, Nocardia, Frankia, Kitasatospora*, and *Streptomyces*. Cytokinins (CK) are also produced by PGPB. Plant totipotent cells are maintained by CK in their shoot and root apical meristems (Howell et al., 2003). In plants, three receptors are responsible for CK signaling, which are CRE1/AHK4/WOL, AHK2, and AHK3. The higher levels of cytokinin are positively correlated with increased plant growth.

Abscisic acid (ABA) is commonly called the stress hormone, and this is upregulated when there is water deficiency under salinity stress conditions in the root zone. The increase in the level of ABA under salinity helps the plant to cope with the impact of stress. ABA helps in the accumulation of compatible solutes such as sugars and proline in root vacuoles, as well as of Ca^2+^ and K^+^, which mitigate the effects of high salinity (Numan et al., 2018). Gibberellins are also produced by bacteria, which helps in the promotion of the growth and yield of plants. Rice roots colonized by *Rhizobium* show higher production of gibberellins and auxins, which leads to increased plant growth and development (Bottini et al., 1989). Naz et al. (2009) reported that when a halotolerant bacterium was inoculated in soybean plant, root and shoot length and biomass were improved through the overproduction of proline, ABA, *trans-*zeatin riboside, GA3, and IAAs. *Rhodococcus* and *Novoshingobium* species have been shown to metabolize ABA *in vitro*, which apparently helps in reducing the levels of ABA in plants. Some of the PGPR help in the production of ABA, while others are shown to metabolize it and produce variable effects under salinity stress (Qin et al., 2016).

### Extracellular Polymeric Substance (EPS) Production

A number of biopolymers, such as polysaccharides, polyamides, and polyesters are produced by microorganisms under natural conditions. A wide spectrum of multifunctional polysaccharides are synthesized, including structural, intracellular, and extracellular or exo-polysaccharides (Haggag, 2010; Verma et al., 2015; Vurukonda et al., 2016; Gupta et al., 2019). EPS-producing PGPB can play a significant role in alleviating salinity stress (Ashraf et al., 2004; Upadhyay et al., 2011) as EPS binds with cations, such as Na^+^, and decreases bioavailable ions for plant uptake. The production of EPS is an important criterion for the classification of stress-tolerant microbes. Most of the bacteria survive under stress conditions due to the production of EPS (Table 1). EPS promotes bacterial survival due to enhancing water retention capacity and regulating the diffusion of organic carbon sources. Bacteria also contain high molecular weight lipopolysaccharide–protein (LP) (carbohydrate complexes) and polysaccharide–lipid (PL) complexes that are responsible for desiccation tolerance. EPS also helps in the establishment of plant-microbe interactions (Ashraf et al., 2004; Haggag, 2010; Vurukonda et al., 2016) by providing a micro-environment in which microbes can survive under stress conditions. It also helps bacteria to attach to the plant and colonize it in response to root exudates. The composition and concentration of EPS changes dramatically under drought and salinity stress conditions. EPS is secreted in soil by microbes in the form of slime material and binds with soil due to Van der Waals forces, hydrogen bonding, cation bridges, and anion adsorption mechanisms (Naseem and Bano, 2014; Ansari et al., 2019). Thus, slime material forms a protective capsule around soil aggregates, and when plants are inoculated with EPS producing microbes, it displays resistance against salinity. Production of EPS by soil microbes around roots also increases water potential and uptake of nutrients by plants (Ashraf et al., 2004; Naseem and Bano, 2014).

The formation of biofilm is a common property of microbes under salinity stress. Biofilm is an aggregate of microbes in which they adhere to each other and protect themselves from adverse effects. Recent studies suggest that EPS modulates the physical and chemical attributes of microbes under saline conditions (Haggag, 2010). EPS plays an important role in maintaining the structural stability of the biofilms (Zhang et al., 2011; Zheng et al., 2016). The types of substances secreted determine the strength of these biofilms against salinity stress. High salinity can lead to disruption of biofilms produced through effects on the microbial metabolism and physiological processes (Bassin et al., 2011; Li et al., 2018). *Curtobacterium albidum* strain SRV4 alleviated salinity stress in rice plant due to production of EPS in addition to nitrogen (N_2_) fixation, IAA production, and ACC deaminase activity (Vimal et al., 2019).

### Induction of Synthesis of Plant Osmolytes and Antioxidant Activity

Organic osmolytes produced by bacteria include highly soluble organic compounds, such as sugars, sugar alcohols, glucosyl glycerol, betaines, amino acids, and tetrahydropyrimidine (Ciulla et al., 1997). Organic solutes accumulated in the bacterial cytoplasm may or may not be synthesized by bacteria. Sometimes the organic osmolytes are taken up from the environment (Ciulla et al., 1997). Likewise, in plants, osmolyte accumulation takes place to combat salinity stress. The accumulation of osmolytes in the cytoplasm helps to maintain the osmotic balance of the cell. Major plant osmolytes include di- and oligo-saccharides, sugar alcohols, glycine, betaine, proline, and glutamate (Chen and Jiang, 2010; Suprasanna et al., 2016). Sugars, primarily disaccharides such as sucrose, trehalose, oligosaccharides raffinose, and fructans, act as osmoprotectants and are major drivers in the plant stress tolerance mechanism. Apart from their osmoprotective functions, these organic osmolytes carry out several other vital functions, such as helping the survival of the microbiome inhabiting the plants. Sucrose accumulation is associated with the survival of *Craterostigma plantagineum* under plant tissue dehydration (Norwood et al., 2000). Sugar alcohols such as pinitol, mannitol, myo-inositol ononitol, and sorbitol act as osmoregulators during salinity stress and also act as signaling molecules (Slama et al., 2015; Suprasanna et al., 2016). Amino acids such as proline, betaine, and γ-aminobutyric acid are some of the organic osmolytes. Proline accumulation is observed in plant members of the *Aizoaceae* family and also most of the halophytes. DMSP (Dimethyl sulfoniopropionate) also acts as an osmoprotectant (Suprasanna et al., 2016).

Enzymatic and non-enzymatic antioxidants play a major role in the defense mechanism through regulation of ROS levels. The important enzymatic antioxidants include CAT, guaiacol peroxidase, SOD, APX, monodehydro-ascorbate reductase, dehydrogenase ascorbate, and glutathione reductase. Non-enzymatic antioxidant compounds comprise ascorbate, glutathione, carotenoids, tocopherols, flavonoids, etc. (Sengupta et al., 2016).

### Essential Nutrient Uptake

A low level of essential nutrients is one of the major causes of the reduction of plant growth and productivity. Salinity reduces the uptake and accumulation of essential plant nutrients such as nitrogen (N), phosphorus (P), and potassium (K^+^) and water due to high osmotic pressure and ion toxicity. Besides, plants need extra nutrients for maintenance of growth under stress conditions (Meena et al., 2014; Abbas et al., 2015; Sharma and Archana, 2016). Crop yield is also known to be affected in saline soils due to effects on the nutrient uptake and translocation (Nautiyal et al., 2008; Shi-Ying et al., 2018). Plant-associated PGPB are known to improved nutrient uptake and accumulation in many ways (Jaiswal et al., 2016).

Nitrogen, an important nutrient element required for plant growth, is limited under salinity stress. The inoculation of PGPB increases N uptake by symbiotic and non-symbiotic association with the plants (Santi et al., 2013). Phosphorus is the second important element after N and is taken up by roots in monobasic (H_2_PO_4_^–^) or dibasic (HPO_4_^2–^) soluble forms. In natural conditions, the majority of P is present in inorganic and organic forms in soils and is mostly unavailable to plants. PGPB convert the unavailable P forms into available ones by acidification and chelation (Etesami et al., 2014). Potassium has a significant role in the growth and development of the plant. To achieve maximum yield, K is needed in adequate quantities (50–300 kg ha^–1^) by all crops. However, most of the K of soil is not directly available for plant uptake. Moreover, K availability to plants decreases due to salinity stress. In this situation, K-solubilizing bacteria (KSB) are an effective tool to fulfill the K requirement of crops (Mukherjee et al., 2019). It is reported that KSB convert mineral K into available K for plant uptake (Etesami et al., 2017; Vasanthi et al., 2018). The rhizobacterium *Burkholderia* releases K from minerals in soil (Kang et al., 2014a). A study by Mayak et al. (2004) showed increased P and K uptake by tomato plants when inoculated with *Achromobacter piechaudii.* The NPK contents of the wheat leaves increased significantly under salt stress conditions upon inoculation with *Bacillus aquimaris* (Upadhyay and Singh, 2015).

Plant growth-promoting bacteria also release/increase the availability of mineral elements like Cu, Fe, Mn, Zn, etc., to plants by chelation and acidification of soil (Etesami et al., 2014). Siderophore production is a major feature of PGPB. Iron is the second most abundant metal in the Earth’s crust. It is essential for certain iron-sulfur complex enzymes and iron-containing proteins and plays a major role in plant growth by participating in the synthesis of chlorophyll. Salinity enhances Fe-related deficiency, i.e., chlorosis in plants. The Fe availability is also reduced in saline conditions because of the inhibition of the proton pumps. Siderophore-producing bacterial strains have a higher affinity toward Fe than phytosiderophores, and thus they can remove Fe from Fe^3^-phytosiderophore complex. Studies have suggested that microbial activity plays a major role in Fe accumulation in roots and also its transport to leaves (Masalha et al., 2000). A study by Rungin et al. (2012) showed that the siderophore-producing endophytes *Streptomyces* increased root and shoot biomass due to enhanced supply of Fe. Siderophore producing-PGPB have been shown to impart salt tolerance in several plant models (Kavamura et al., 2013; Ramadoss et al., 2013). The increased root exudates owing to PGPB-induced root growth can also, in turn, increase the availability of nutrients such as P and micronutrients (Kang et al., 2014a).

## Role of the Phytomicrobiome in Salinity Stress Tolerance

**FIGURE 6:**
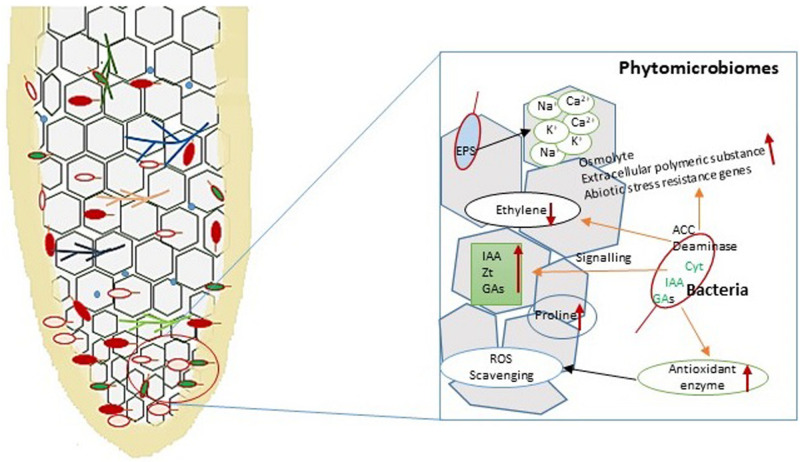
Diagrammatic representations of plant–microbe interaction in the root and their different functions under salinity stress. Bacteria produce signaling molecules that help to support plant growth and development under stress conditions. Under salinity stress, bacterial the enzyme ACC deaminase reduces ethylene synthesis and enhances indole-3-acetic acid (IAA), Zeatin (Zt), and gibberellins (GA) in the plant. Exopolysaccharides (EPS) secreted by bacteria bind with different ions (Ca^2+^, K^+^, and Na^+^) to prevent their effects on the plant. Endophytic bacterial strains induce antioxidants for controlling reactive oxygen species (ROS) generation in the plant under abiotic stress conditions.

Endophytic PGPB employ mechanisms similar to those used by rhizosphere microbes in supporting plant growth and in imparting stress tolerance (Figure 6). The microbial communities inhabiting inside or on the surface of the plant are called the phytomicrobiome. All of the microbes in a certain phytomicrobiome may not be required at a given time. The phytomicrobiome also plays an important role in the interaction of plants with microbial communities of the rhizoplane, rhizosphere, and phyllosphere (Singh and Trivedi, 2017). The microbial communities associated with the plants may inhabit different plant parts. The microbial communities associated with different parts of plant are known as the root, shoot, leaf, flower, and seed microbiome (Qin et al., 2016). The root microbiome of the plant is crucial in determining the survival of the plant under conditions of stress, including salinity and drought stresses. Bacteria isolated from extreme environmental conditions have been shown to exhibit salinity stress resistance properties. *P. fluorescens* were isolated from rhizosphere soil of the Saharan region and showed a PGPB property in maize under salinity stress (Zerrouk et al., 2016). Several microbes inhabiting plants growing under high salt conditions also show adaptations toward salinity stress and thrive well in such conditions. These are called the halophyte microbiome. A major adaptation of halophilic microbes is that they maintain the protein structure and enzymatic activity for various metabolic processes even at high salt concentrations (Ruppel et al., 2013).

The basic mechanism by which salinity-tolerant microbes thrive in saline habitats is by avoiding high salt concentrations inside the cytoplasm. This is achieved through modifications in the cell wall construction in which specific membrane proteins, lipids, and exopolysaccharides are formed. Another method of avoiding high salt concentration inside the cytoplasm is by pumping ions out of the cell. Na^+^/H^+^ antiporters such as NhaA, an antiporter of *Escherichia coli* and many enterobacteria (Hunte et al., 2005), help in pumping out excess Na^+^ (Ruppel et al., 2013). Other adaptations for survival under high salinity conditions include the development of proteins and enzymes capable of performing metabolic functions. Some microbes also develop organic osmolytes, which accumulate in the cytoplasm to make them resistant against osmotic pressure under high salinity stress. The organic osmolytes are also called compatible solutes as they provide resistance to different molar concentrations of salinity stress by maintaining a suitable molar concentration in the cytoplasm (Kunte, 2006).

## Future Perspectives and Conclusion

Increasing salinity is posing serious threats to agricultural productivity, making it a matter of concern for policymakers and agriculture scientists. Salinity stress is also suppressing beneficial microbes in the soil. Understanding the complexity of plant-soil-microbe interactions under salinity stress offers novel possibilities for employing potential PGPBs as a sustainable tool for achieving high crop productivity. However, despite significant research already having been done in this area, there is a lack of knowledge on some important aspects. Knowledge regarding communication (signaling) between plants and microbes is still limited. Besides, the mechanisms of growth promotion in the presence of PGPB under salinity stress still need to be delineated in further detail up to the gene level. There is also a need to understand the role of epigenetic processes in salinity stress tolerance in plants and to delineate the functional interplay of PGPB. Further research should be focused on the utilization of effective salt-tolerant PGPB in salt-affected agricultural fields to promote the development of bacterial inoculants as commercial biofertilizers for improving salinity tolerance and crop productivity.

The combined use of rhizosphere PGPB and endophytic microbiomes may have a synergistic effect in alleviating salinity stress and for sustainably enhancing agricultural productivity. Overall, the future research should be focused on: (1) exploring the mechanisms of cross-talk between PGPB and plants in salinity stress environments, (2) understanding the mechanisms by which salt tolerance is conferred to plants by PGPB, (3) identifying the genes involved in plant growth promotion under salinity stress in natural environments, (4) transferring the identified genes through biotechnology into crop plants, (5) exploring the combined application of salt-tolerant PGPB and mycorrhizae under natural conditions, and (6) promoting the role of PGPB as a bio-fertilizer for sustainable agricultural production.

## Author Contributions

AK and SS wrote the manuscript. AG created the table, and reference setting. JV edited the manuscript. All authors contributed to the article and approved the submitted version.

## Conflict of Interest

The authors declare that the research was conducted in the absence of any commercial or financial relationships that could be construed as a potential conflict of interest.

## References

[B1] Abbas T. Balal R. M. Shahid M. A. Pervez M. A. Ayyub C. M. Aqueel M. A. (2015). Silicon-induced alleviation of NaCl toxicity in okra (Abelmoschus esculentus) is associated with enhanced photosynthesis, osmoprotectants and antioxidant metabolism. *Acta Physiol. Plant.* 37:6 10.1007/s11738-014-1768-5

[B2] Abdel-Aal H. K. Zohdy K. M. Kareem M. A. (2010). Hydrogen production using sea water electrolysis. *TOFCJ* 3:1 10.2174/1875932701003010001

[B3] Ahmad M. Zahir Z. A. Asghar H. N. Asghar M. (2011). Inducing salt tolerance in mung bean through co-inoculation with rhizobia and plant-growth-promoting rhizobacteria containing 1-aminocyclopropane-1-carboxylate deaminase. *Can. J. Microbiol.* 57 578–589. 10.1139/w11-044 21770816

[B4] Ali S. Charles T. C. Glick B. R. (2014). Amelioration of high salinity stress damage by plant growth-promoting bacterial endophytes that contain ACC deaminase. *Plant Physiol. Biochem.* 80 160–167. 10.1016/j.plaphy.2014.04.003 24769617

[B5] Allison S. D. Martiny J. B. (2008). Resistance, resilience, and redundancy in microbial communities. *Proc. Natl. Acad. Sci. U.S.A.* 105(Suppl. 1), 11512–11519. 10.1073/pnas.0801925105 18695234 PMC2556421

[B6] Ansari F. A. Ahmad I. Pichtel J. (2019). Growth stimulation and alleviation of salinity stress to wheat by the biofilm forming Bacillus pumilus strain FAB10. *Appl. Soil Ecol.* 143 45–54. 10.1016/j.apsoil.2019.05.023

[B7] Ashraf M. Hasnain S. Berge O. Mahmood T. (2004). Inoculating wheat seedlings with exopolysaccharide-producing bacteria restricts sodium uptake and stimulates plant growth under salt stress. *Biol. Fertil. Soils* 40 157–162. 10.1007/s00374-004-0766-y

[B8] Ayers R. S. Westcot D. W. (1985). *Water Quality for Agriculture.* (Vol. 29). Rome: Food and Agriculture Organization of the United Nations.

[B9] Bano A. Fatima M. (2009). Salt tolerance in *Zea mays* (L). following inoculation with Rhizobium and Pseudomonas. *Biol. Fertil. Soils* 45 405–413. 10.1007/s00374-008-0344-9

[B10] Barnawal D. Bharti N. Maji D. Chanotiya C. S. Kalra A. (2014). ACC deaminase-containing Arthrobacter protophormiae induces NaCl stress tolerance through reduced ACC oxidase activity and ethylene production resulting in improved nodulation and mycorrhization in *Pisum sativum*. *J. Plant. Physiol.* 171 884–894. 10.1016/j.jplph.2014.03.007 24913045

[B11] Barnawal D. Bharti N. Pandey S. S. Pandey A. Chanotiya C. S. Kalra A. (2017). Plant growthpromoting rhizobacteria enhance wheat salt and drought stress tolerance by altering endogenous phytohormone levels and TaCTR1/TaDREB2 expression. *Physiol. Plant.* 161 502–514. 10.1111/ppl.12614 28786221

[B12] Bassin J. P. Dezotti M. Sant’Anna G. L. Jr. (2011). Nitrification of industrial and domestic saline wastewaters in moving bed biofilm reactor and sequencing batch reactor. *J. Hazard. Mater.* 185 242–248. 10.1016/j.jhazmat.2010.09.024 20933327

[B13] Bharti N. Pandey S. S. Barnawal D. Patel V. K. Kalra A. (2016). Plant growth promoting rhizobacteria Dietzia natronolimnaea modulates the expression of stress responsive genes providing protection of wheat from salinity stress. *Sci. Rep.* 6:34768. 10.1038/srep34768 27708387 PMC5052518

[B14] Bhise K. K. Bhagwat P. K. Dandge P. B. (2017). Synergistic effect of *Chryseobacterium gleum* sp. SUK with ACC deaminase activity in alleviation of salt stress and plant growth promotion in *Triticum aestivum* L. *3 Biotech.* 7:105. 10.1007/s13205-017-0739-0 28560646 PMC5449284

[B15] Bottini R. Fulchieri M. Pearce D. Pharis R. P. (1989). Identification of gibberellins A1, A3, and iso-A3 in cultures of *Azospirillum lipoferum*. *Plant Physiol.* 90 45–47. 10.1104/pp.90.1.45 16666766 PMC1061674

[B16] Carter D. L. (1975). “Problems of salinity in agriculture,” in *Plants in Saline Environments*, eds Poljakoff-Mayber A. Gale J. (Berlin: Springer), 25–35. 10.1007/978-3-642-80929-3_3

[B17] Chauhan P. S. Lata C. Tiwari S. Chauhan A. S. Mishra S. K. Agrawal L. (2019). Transcriptional alterations reveal Bacillus amyloliquefaciens-rice cooperation under salt stress. *Sci. Rep.* 9 1–13. 10.1038/s41598-019-48309-8 31417134 PMC6695486

[B18] Chen H. Jiang J. G. (2010). Osmotic adjustment and plant adaptation to environmental changes related to drought and salinity. *Environ. Rev.* 18 309–319. 10.1139/A10-014

[B19] Cheng Z. Park E. Glick B. R. (2007). 1-Aminocyclopropane-1-carboxylate deaminase from *Pseudomonas putida* UW4 facilitates the growth of canola in the presence of salt. *Can. J. Microbiol.* 53 912–918. 10.1139/W07-050 17898846

[B20] Ciulla R. A. Diaz M. R. Taylor B. F. Roberts M. F. (1997). Organic osmolytes in aerobic bacteria from mono lake, an alkaline, moderately hypersaline environment. *Appl. Environ. Microbiol.* 63 220–226. 10.1128/aem.63.1.220-226.1997 16535487 PMC1389101

[B21] Central Soil Salinity Research Institute [CSSRI] (2019). *Extent and Distribution of Salt-affected Soils in India.* Available online at: http://cssri.res.in/index.php/extent-and-distribution-of-salt-affected-soils-in-india (accessed August 24, 2019).

[B22] Daliakopoulos I. N. Tsanis I. K. Koutroulis A. Kourgialas N. N. Varouchakis A. E. Karatzas G. P. (2016). The threat of soil salinity: a European scale review. *Sci. Total Environ.* 573 727–739. 10.1016/j.scitotenv.2016.08.177 27591523

[B23] Das P. Behera B. K. Meena D. K. Azmi S. A. Chatterjee S. Meena K. (2015). Salt stress tolerant genes in halophilic and halotolerant bacteria: paradigm for salt stress adaptation and osmoprotection. *Int. J. Curr. Microbiol. App. Sci.* 4 642–658.

[B24] Deinlein U. Stephan A. B. Horie T. Luo W. Xu G. Schroeder J. I. (2014). Plant salt-tolerance mechanisms. *Trends Plant Sci.* 19 371–379. 10.1016/j.tplants.2014.02.001 24630845 PMC4041829

[B25] Dietz K. J. Vogel M. O. Viehhauser A. (2010). AP2/EREBP transcription factors are part of gene regulatory networks and integrate metabolic, hormonal and environmental signals in stress acclimation and retrograde signalling. *Protoplasma* 245 3–14. 10.1007/s00709-010-0142-8 20411284

[B26] Eakins B. W. Sharman G. F. (2010). *Volumes of the World’s Oceans from ETOPO1.* Boulder, CO: NOAA National Geophysical Data Center.

[B27] Egamberdieva D. Jabborova D. Hashem A. (2015). *Pseudomonas* induces salinity tolerance in cotton (*Gossypium hirsutum*) and resistance to Fusarium root rot through the modulation of indole-3-acetic acid. *Saudi J. Biol. Sci.* 22 773–779. 10.1016/j.sjbs.2015.04.019 26587006 PMC4625397

[B28] Erice G. Ruíz-Lozano J. M. Zamarreño ÁM. García-Mina J. M. Aroca R. (2017). Transcriptomic analysis reveals the importance of JA-Ile turnover in the response of Arabidopsis plants to plant growth promoting rhizobacteria and salinity. *Environ. Exp. Bot.* 143 10–19. 10.1016/j.envexpbot.2017.08.006

[B29] Etesami H. Emami S. Alikhani H. A. (2017). Potassium solubilizing bacteria (KSB): mechanisms, promotion of plant growth, and future prospects A review. *J. Soil Sci. Plant Nutr.* 17 897–911. 10.4067/S0718-95162017000400005 27315006

[B30] Etesami H. Mirseyed Hosseini H. Alikhani H. A. (2014). In planta selection of plant growth promoting endophytic bacteria for rice (*Oryza sativa* L.). *J. Soil Sci. Plant Nutr.* 14 491–503. 10.4067/S0718-95162014005000039 27315006

[B31] FAO (2019). Available online at: http://www.fao.org/soils-portal/soil-management/management-of-some-problem-soils/salt-affected-soils/more-information-on-salt-affected-soils/en (accessed August 24, 2019).

[B32] Fasciglione G. Casanovas E. M. Yommi A. Sueldo R. J. Barassi C. A. (2012). Azospirillum improves lettuce growth and transplant under saline conditions. *J. Sci. Food Agr.* 92 2518–2523. 10.1002/jsfa.5661 22473714

[B33] Ferreira M. J. Silva H. Cunha A. (2019). Siderophore-producing rhizobacteria as a promising tool for empowering plants to cope with iron limitation in saline soils: a review. *Pedosphere* 29 409–420. 10.1016/S1002-0160(19)60810-6

[B34] Gill S. S. Tuteja N. (2010). Reactive oxygen species and antioxidant machinery in abiotic stress tolerance in crop plants. *Plant Physiol. Biochem.* 48 909–930. 10.1016/j.plaphy.2010.08.016 20870416

[B35] Goel D. Singh A. K. Yadav V. Babbar S. B. Murata N. Bansal K. C. (2011). Transformation of tomato with a bacterial codA gene enhances tolerance to salt and water stresses. *J. Plant Physiol.* 168 1286–1294. 10.1016/j.jplph.2011.01.010 21342716

[B36] Golldack D. Lüking I. Yang O. (2011). Plant tolerance to drought and salinity: stress regulating transcription factors and their functional significance in the cellular transcriptional network. *Plant Cell Rep.* 30 1383–1391. 10.1007/s00299-011-1068-0 21476089

[B37] Gupta B. Huang B. (2014). Mechanism of salinity tolerance in plants: physiological, biochemical, and molecular characterization. *Int. J. Genomics.* 2014:701596. 10.1155/2014/701596 24804192 PMC3996477

[B38] Gupta J. Rathour R. Singh R. Thakur I. S. (2019). Production and characterization of extracellular polymeric substances (EPS) generated by a carbofuran degrading strain *Cupriavidus* sp. ISTL7. *Bioresour. Technol.* 282 417–424. 10.1016/j.biortech.2019.03.054 30884462

[B39] Haggag W. M. (2010). The role of biofilm exopolysaccharides on biocontrol of plant diseases. *Biopolymers* 14 271–284. 10.5772/10266

[B40] Halo B. A. Khan A. L. Waqas M. Al-Harrasi A. Hussain J. Ali L. (2015). Endophytic bacteria (*Sphingomonas* sp. LK11) and gibberellin can improve *Solanum lycopersicum* growth and oxidative stress under salinity. *J. Plant Interact.* 10 117–125. 10.1080/17429145.2015.1033659

[B41] Hardy N. Shainberg I. Gal M. Keren R. (1983). The effect of water quality and storm sequence upon infiltration rate and crust formation. *J. Soil Sci.* 34 665–676. 10.1111/j.13652389.1983.tb01063.x

[B42] Howell S. H. Lall S. Che P. (2003). Cytokinins and shoot development. *Trends Plant Sci.* 8 453–459. 10.1016/S1360-1385(03)00191-2 13678913

[B43] Hunte C. Screpanti E. Venturi M. Rimon A. Padan E. Michel H. (2005). Structure of a Na+/H+ antiporter and insights into mechanism of action and regulation by pH. *Nature* 435 1197–1202. 10.1038/nature03692 15988517

[B44] Jaiswal D. K. Verma J. P. Prakash S. Meena V. S. Meena R. S. (2016). “Potassium as an important plant nutrient in sustainable agriculture: a state of the art,” in *Potassium Solubilizing Microorganisms for Sustainable Agriculture*, eds Meena V. S. Maurya B. R. Verma J. P. Meena R. S. (New Delhi: Springer), 21–29. 10.1007/978-81-322-2776-2_2

[B45] Jha Y. Subramanian R. B. (2014). PGPR regulate caspase-like activity, programmed cell death, and antioxidant enzyme activity in paddy under salinity. *Physiol. Mol. Biol. Plants* 20 201–207. 10.1007/s12298-014-0224-8 24757324 PMC3988331

[B46] Jha Y. Subramanian R. B. Patel S. (2011). Combination of endophytic and rhizospheric plant growth promoting rhizobacteria in *Oryza sativa* shows higher accumulation of osmoprotectant against saline stress. *Acta Physiol. Plant* 33 797–802. 10.1007/s11738-010-0604-9

[B47] Joshi R. Paul M. Kumar A. Pandey D. (2019). Role of calreticulin in biotic and abiotic stress signalling and tolerance mechanisms in plants. *Gene* 714:144004. 10.1016/j.gene.2019.144004 31351124

[B48] Kalaji H. M. Bosa K. Kościelniak J. Żuk-Gołaszewska K. (2011). Effects of salt stress on photosystem II efficiency and CO2 assimilation of two Syrian barley landraces. *Environ. Exp. Bot.* 73 64–72. 10.1016/j.envexpbot.2010.10.009

[B49] Kang S.-M. Khan A. L. Waqas M. You Y.-H. Kim J.-H. Kim J.-G. (2014a). Plant growth-promoting rhizobacteria reduce adverse effects of salinity and osmotic stress by regulating phytohormones and antioxidants in *Cucumis sativus*. *J. Plant Interact.* 9 673–682. 10.1080/17429145.2014.894587

[B50] Kang S. M. Radhakrishnan R. Khan A. L. Kim M. J. Park J. M. Kim B. R. (2014b). Gibberellin secreting rhizobacterium, *Pseudomonas putida* H-2-3 modulates the hormonal and stress physiology of soybean to improve the plant growth under saline and drought conditions. *Plant Physiol. Biochem.* 84 115–124. 10.1016/j.plaphy.2014.09.001 25270162

[B51] Kavamura V. N. Santos S. N. da Silva J. L. Parma M. M. Ávila L. A. Visconti A. (2013). Screening of Brazilian cacti rhizobacteria for plant growth promotion under drought. *Microbiol. Res.* 168 183–191. 10.1016/j.micres.2012.12.002 23279812

[B52] Khan A. Zhao X. Q. Javed M. T. Khan K. S. Bano A. Shen R. F. (2016). Bacillus pumilus enhances tolerance in rice (*Oryza sativa* L.) to combined stresses of NaCl and high boron due to limited uptake of Na+. *Environ. Exp. Bot.* 124 120–129. 10.1016/j.envexpbot.2015.12.011

[B53] Kim K. Jang Y. J. Lee S. M. Oh B. T. Chae J. C. Lee K. J. (2014). Alleviation of salt stress by *Enterobacter* sp. EJ01 in tomato and *Arabidopsis* is accompanied by up-regulation of conserved salinity responsive factors in plants. *Mol. Cells.* 37:109. 10.14348/molcells.2014.2239 24598995 PMC3935623

[B54] Kırtel O. Versluys M. Van den Ende W. Öner E. T. (2018). Fructans of the saline world. *Biotechnol. Adv.* 36 1524–1539. 10.1016/j.biotechadv.2018.06.009 29935267

[B55] Kohler J. Hernández J. A. Caravaca F. Roldán A. (2009). Induction of antioxidant enzymes is involved in the greater effectiveness of a PGPR versus AM fungi with respect to increasing the tolerance of lettuce to severe salt stress. *Environ. Exp. Bot.* 65 245–252. 10.1016/j.envexpbot.2008.09.008

[B56] Kumar A. Verma J. P. (2018). Does plant—Microbe interaction confer stress tolerance in plants: a review? *Microbiol. Res.* 207 41–52. 10.1016/j.micres.2017.11.004 29458867

[B57] Kumar A. Verma J. P. (2019). “The role of microbes to improve crop productivity and soil health,” in *Ecological Wisdom Inspired Restoration Engineering*, eds Achal V. Mukherjee A. (Singapore: Springer), 249–265. 10.1007/978-981-13-0149-0_14

[B58] Kumar J. Singh S. Singh M. Srivastava P. K. Mishra R. K. Singh V. P. (2017). Transcriptional regulation of salinity stress in plants: a short review. *Plant Gene* 11 160–169. 10.1016/j.plgene.2017.04.001

[B59] Kumar V. Kumar P. Khan A. (2020). Optimization of PGPR and silicon fertilization using response surface methodology for enhanced growth, yield and biochemical parameters of French bean (*Phaseolus vulgaris* L.) under saline stress. *Biocatal. Agric. Biotechnol.* 23:101463 10.1016/j.bcab.2019.101463

[B60] Kunte H. J. (2006). Osmoregulation in bacteria: compatible solute accumulation and osmosensing. *Environ. Chem.* 3 94–99. 10.1071/EN06016

[B61] Li J. Ye W. Wei D. Ngo H. H. Guo W. Qiao Y. (2018). System performance and microbial community succession in a partial nitrification biofilm reactor in response to salinity stress. *Bioresour. Technol.* 270 512–518. 10.1016/j.biortech.2018.09.068 30248650

[B62] Liu S. Hao H. Lu X. Zhao X. Wang Y. Zhang Y. (2017). Transcriptome profiling of genes involved in induced systemic salt tolerance conferred by *Bacillus amyloliquefaciens* FZB42 in *Arabidopsis thaliana*. *Sci. Rep.* 7:10795. 10.1038/s41598-017-11308-8 28904348 PMC5597682

[B63] Mahmuduzzaman M. Ahmed Z. U. Nuruzzaman A. K. M. Ahmed F. R. S. (2014). Causes of salinity intrusion in coastal belt of Bangladesh. *Int. J. Plant Res.* 4 8–13. 10.5923/s.plant.201401.02 22499009

[B64] Masalha J. Kosegarten H. Elmaci Ö Mengel K. (2000). The central role of microbial activity for iron acquisition in maize and sunflower. *Biol. Fert. Soils.* 30 433–439. 10.1007/s003740050021

[B65] Mayak S. Tirosh T. Glick B. R. (2004). Plant growth-promoting bacteria confer resistance in tomato plants to salt stress. *Plant Physiol. Biochem.* 42 565–572. 10.1016/j.plaphy.2004.05.009 15246071

[B66] Mbodj D. Effa-Effa B. Kane A. Manneh B. Gantet P. Laplaze L. (2018). Arbuscular mycorrhizal symbiosis in rice: establishment, environmental control and impact on plant growth and resistance to abiotic stresses. *Rhizosphere* 8 12–26. 10.1016/j.rhisph.2018.08.003

[B67] Meena V. S. Maurya B. R. Verma J. P. (2014). Does a rhizospheric microorganism enhance K+ availability in agricultural soils? *Microbiol. Res.* 169 337–347. 10.1016/j.micres.2013.09.003 24315210

[B68] Mehta P. Jajoo A. Mathur S. Bharti S. (2010). Chlorophyll a fluorescence study revealing effects of high salt stress on Photosystem II in wheat leaves. *Plant Physiol. Biochem.* 48 16–20. 10.1016/j.plaphy.2009.10.006 19932973

[B69] Miller G. A. D. Suzuki N. CiftciYilmaz S. U. L. T. A. N. Mittler R. O. N. (2010). Reactive oxygen species homeostasis and signalling during drought and salinity stresses. *Plant Cell Environ.* 33 453–467. 10.1111/j.1365-3040.2009.02041.x 19712065

[B70] Mukherjee A. Gaurav A. K. Singh S. Chouhan G. K. Kumar A. Das S. (2019). Role of Potassium (K) Solubilising Microbes (KSM) in growth and induction of resistance against biotic and abiotic stress in plant: a book review. *Clim. Change Environ. Sustain.* 7 212–214.

[B71] Narayana D. V. Babu R. (1983). Estimation of soil erosion in India. *J. Irrig. Drain Eng.* 109 419–434. 10.1061/(ASCE)0733-94371983109:4(419)

[B72] Naseem H. Bano A. (2014). Role of plant growth-promoting rhizobacteria and their exopolysaccharide in drought tolerance of maize. *J. Plant Interact.* 9 689–701. 10.1080/17429145.2014.902125

[B73] Nathawat N. S. Kuhad M. S. Goswami C. L. Patel A. L. Kumar R. (2005). Nitrogen-metabolizing enzymes: effect of nitrogen sources and saline irrigation. *J. Plant Nutr.* 28 1089–1101. 10.1081/PLN-200058911

[B74] Nautiyal C. S. Srivastava S. Chauhan P. S. (2008). “Rhizosphere colonization: molecular determinants from plant-microbe coexistence perspective,” in *Molecular Mechanisms of Plant and Microbe Coexistence*, eds Nautiyal C. S. Dion P. (Berlin: Springer), 99–123. 10.1007/978-3-540-75575-3_4

[B75] Nautiyal C. S. Srivastava S. Chauhan P. S. Seem K. Mishra A. Sopory S. K. (2013). Plant growth-promoting bacteria Bacillus amyloliquefaciens NBRISN13 modulates gene expression profile of leaf and rhizosphere community in rice during salt stress. *Plant Physiol. Biochem.* 66 1–9. 10.1016/j.plaphy.2013.01.020 23454292

[B76] Naz I. Bano A. Ul-Hassan T. (2009). Isolation of phytohormones producing plant growth promoting rhizobacteria from weeds growing in Khewra salt range, Pakistan and their implication in providing salt tolerance to *Glycine max* L. *Afr. J. Biotechnol.* 8 5762–5768. 10.5897/AJB09.1176

[B77] Nia S. H. Zarea M. J. Rejali F. Varma A. (2012). Yield and yield components of wheat as affected by salinity and inoculation with *Azospirillum* strains from saline or non-saline soil. *J. Saudi Soc. Agric. Sci.* 11 113–121. 10.1016/j.jssas.2012.02.001

[B78] Norwood M. Truesdale M. R. Richter A. Scott P. (2000). Photosynthetic carbohydrate metabolism in the resurrection plant *Craterostigma plantagineum*. *J. Exp. Bot.* 51 159–165. 10.1093/jexbot/51.343.159 10938822

[B79] Numan M. Bashir S. Khan Y. Mumtaz R. Shinwari Z. K. Khan A. L. (2018). Plant growth promoting bacteria as an alternative strategy for salt tolerance in plants: a review. *Microbiol. Res.* 209 21–32. 10.1016/j.micres.2018.02.003 29580619

[B80] Palaniyandi S. A. Damodharan K. Yang S. H. Suh J. W. (2014). *Streptomyces* sp. strain PGPA39 alleviates salt stress and promotes growth of ‘Micro Tom’tomato plants. *J. Appl. Microbiol.* 117 766–773. 10.1111/jam.12563 24909841

[B81] Panwar M. Tewari R. Nayyar H. (2016). Native halo-tolerant plant growth promoting rhizobacteria *Enterococcus* and *Pantoea* sp. improve seed yield of Mung bean (*Vigna radiata* L.) under soil salinity by reducing sodium uptake and stress injury. *Physiol. Mol. Biol. Plants* 22 445–459. 10.1007/s12298-016-0376-9 27924118 PMC5120033

[B82] Qin Y. Druzhinina I. S. Pan X. Yuan Z. (2016). Microbially mediated plant salt tolerance and microbiome-based solutions for saline agriculture. *Biotechnol. Adv.* 34 1245–1259. 10.1016/j.biotechadv.2016.08.005 27587331

[B83] Ramadoss D. Lakkineni V. K. Bose P. Ali S. Annapurna K. (2013). Mitigation of salt stress in wheat seedlings by halotolerant bacteria isolated from saline habitats. *Springer Plus* 2:6. 10.1186/2193-1801-2-6 23449812 PMC3579424

[B84] Rangarajan S. Saleena L. M. Nair S. (2002). Diversity of *Pseudomonas* spp. isolated from rice rhizosphere populations grown along a salinity gradient. *Microb. Ecol.* 43 280–289. 10.1007/s00248-002-2004-1 12023735

[B85] Rungin S. Indananda C. Suttiviriya P. Kruasuwan W. Jaemsaeng R. Thamchaipenet A. (2012). Plant growth enhancing effects by a siderophore-producing endophytic streptomycete isolated from a Thai jasmine rice plant (*Oryza sativa* L. cv. KDML105). *Antonie Van Leeuwenhoek* 102 463–472. 10.1007/s10482-012-9778-z 22836676

[B86] Ruppel S. Franken P. Witzel K. (2013). Properties of the halophyte microbiome and their implications for plant salt tolerance. *Funct. Plant Biol.* 40 940–951. 10.1071/FP12355 32481163

[B87] Sahoo R. K. Ansari M. W. Pradhan M. Dangar T. K. Mohanty S. Tuteja N. (2014). A novel *Azotobacter vinellandii* (SRI Az 3) functions in salinity stress tolerance in rice. *Plant Signal. Behav.* 9 511–523. 10.4161/psb.29377 25763502 PMC4203646

[B88] Santi C. Bogusz D. Franche C. (2013). Biological nitrogen fixation in non-legume plants. *Ann. Bot.* 111 743–767. 10.1093/aob/mct048 23478942 PMC3631332

[B89] Sapre S. Gontia-Mishra I. Tiwari S. (2018). *Klebsiella* sp. confers enhanced tolerance to salinity and plant growth promotion in oat seedlings (*Avena sativa*). *Microbiol. Res.* 206 25–32. 10.1016/j.micres.2017.09.009 29146257

[B90] Saravanakumar D. Samiyappan R. (2007). ACC deaminase from *Pseudomonas fluorescens* mediated saline resistance in groundnut (*Arachis hypogea*) plants. *J. Appl. Microbiol.* 102 1283–1292. 10.1111/j.1365-2672.2006.03179.x 17448163

[B91] Sarkar A. Pramanik K. Mitra S. Soren T. Maiti T. K. (2018). Enhancement of growth and salt tolerance of rice seedlings by ACC deaminase-producing *Burkholderia* sp. MTCC 12259. *J. Plant Physiol.* 231 434–442. 10.1016/j.jplph.2018.10.010 30414570

[B92] Sengupta A. Chakraborty M. Saha J. Gupta B. Gupta K. (2016). “Polyamines: osmoprotectants in plant abiotic stress adaptation,” in *Osmolytes and Plants Acclimation to Changing Environment: Emerging Omics Technologies*, eds Iqbal N. Nazar R. Khan N. A. (New Delhi: Springer), 97–127. 10.1007/978-81-322-2616-1_7

[B93] Sharma R. K. Archana G. (2016). Cadmium minimization in food crops by cadmium resistant plant growth promoting rhizobacteria. *Appl. Soil Ecol.* 107 66–78. 10.1016/j.apsoil.2016.05.009

[B94] Shi-Ying Z. Cong F. Yong-xia W. Yun-sheng X. Wei X. Xiao-Long C. (2018). Salt-tolerant and plant growth-promoting bacteria isolated from high-yield paddy soil. *Can. J. Microbiol.* 64 968–978. 10.1139/cjm-2017-0571 30148967

[B95] Shrivastava P. Kumar R. (2015). Soil salinity: a serious environmental issue and plant growth promoting bacteria as one of the tools for its alleviation. *Saudi J. Biol. Sci.* 22 123–131. 10.1016/j.sjbs.2014.12.001 25737642 PMC4336437

[B96] Shukla P. S. Agarwal P. K. Jha B. (2012). Improved salinity tolerance of *Arachishypogaea* (L.) by the interaction of halotolerant plant-growth-promoting rhizobacteria. *J. Plant Growth Regul.* 31 195–206. 10.1007/s00344-011-9231-y

[B97] Siddiqui M. H. Khan M. N. Mohammad F. Khan M. M. A. (2008). Role of nitrogen and gibberellin (GA3) in the regulation of enzyme activities and in osmoprotectant accumulation in *Brassica juncea* L. under salt stress. *J. Agron. Crop Sci.* 194 214–224. 10.1111/j.1439-037X.2008.00308.x

[B98] Singh B. K. Trivedi P. (2017). Microbiome and the future for food and nutrient. *Secur. Microb. Biotechnol.* 10 50–53. 10.1111/1751-7915.12592 28074557 PMC5270726

[B99] Singh B. K. Trivedi P. Singh S. Macdonald C. A. Verma J. P. (2018). Emerging microbiome technologies for sustainable increase in farm productivity and environmental security. *Microbiol. Aust.* 39 17–23. 10.1071/MA18006

[B100] Singh R. P. Jha P. (2016). Alleviation of salinity-induced damage on wheat plant by an ACC deaminase-producing *halophilic bacterium Serratia* sp. SL-12 isolated from a salt lake. *Symbiosis* 69, 101–111. 10.1007/s13199-016-0387-x

[B101] Singh R. P. Jha P. Jha P. N. (2015). The plant-growth-promoting bacterium Klebsiella sp. SBP-8 confers induced systemic tolerance in wheat (*Triticum aestivum*) under salt stress. *J. Plant Physiol.* 184 57–67. 10.1016/j.jplph.2015.07.002 26217911

[B102] Slama I. Abdelly C. Bouchereau A. Flowers T. Savouré A. (2015). Diversity, distribution and roles of osmoprotective compounds accumulated in halophytes under abiotic stress. *Ann. Bot.* 115 433–447. 10.1093/aob/mcu239 25564467 PMC4332610

[B103] Suarez C. Cardinale M. Ratering S. Steffens D. Jung S. Montoya A. M. Z. (2015). Plant growth-promoting effects of Hartmannibacter diazotrophicus on summer barley (*Hordeum vulgare* L.) *under salt stress*. *Appl. Soil Ecol.* 95 23–30. 10.1016/j.apsoil.2015.04.017

[B104] Suprasanna P. Nikalje G. C. Rai A. N. (2016). “Osmolyte accumulation and implications in plant abiotic stress tolerance,” in *Osmolytes and Plants Acclimation to Changing Environment: Emerging Omics Technologies*, eds Iqbal N. Nazar R. Khan N. A. (New Delhi: Springer), 1–12. 10.1007/978-81-322-2616-1_1

[B105] Tang Y. Bao X. Zhi Y. Wu Q. Yin X. Zeng L. (2019). Overexpression of a MYB family gene, OsMYB6, increases drought and salinity stress tolerance in transgenic rice. *Front. Plant Sci.* 10:168. 10.3389/fpls.2019.00168 30833955 PMC6387972

[B106] Tiwari S. Lata C. Chauhan P. S. Nautiyal C. S. (2016). Pseudomonas putida attunes morphophysiological, biochemical and molecular responses in *Cicer arietinum* L. during drought stress and recovery. *Plant Physiol. Biochem.* 99 108–117. 10.1016/j.plaphy.2015.11.001 26744996

[B107] Ullah S. Bano A. (2015). Isolation of plant-growth-promoting rhizobacteria from rhizospheric soil of halophytes and their impact on maize (*Zea mays* L.) under induced soil salinity. *Can. J. Microbiol.* 61 307–313. 10.1139/cjm-2014-0668 25776270

[B108] Upadhyay S. K. Singh D. P. (2015). Effect of salttolerant plant growthpromoting rhizobacteria on wheat plants and soil health in a saline environment. *Plant Biol.* 17 288–293. 10.1111/plb.12173 24750405

[B109] Upadhyay S. K. Singh J. S. Saxena A. K. Singh D. P. (2012). Impact of PGPR inoculation on growth and antioxidant status of wheat under saline conditions. *Plant Biol.* 14 605–611. 10.1111/j.1438-8677.2011.00533.x 22136617

[B110] Upadhyay S. K. Singh J. S. Singh D. P. (2011). Exopolysaccharide-producing plant growth-promoting rhizobacteria under salinity condition. *Pedosphere* 21 214–222. 10.1016/S1002-0160(11)60120-3

[B111] Vacheron J. Desbrosses G. Bouffaud M. L. Touraine B. Moënne-Loccoz Y. Muller D. (2013). Plant growth-promoting rhizobacteria and root system functioning. *Front. Plant Sci.* 4:356. 10.3389/fpls.2013.00356 24062756 PMC3775148

[B112] Vasanthi N. Saleena L. M. Raj S. A. (2018). Silica solubilization potential of certain bacterial species in the presence of different silicate minerals. *Silicon* 10 267–275. 10.1007/s12633-016-9438-4

[B113] Verma J. P. Jaiswal D. K. Meena V. S. Kumar A. Meena R. S. (2015). Issues and challenges about sustainable agriculture production for management of natural resources to sustain soil fertility and health. *J. Clean. Prod.* 107 793–794. 10.1016/j.jclepro.2015.04.130

[B114] Vimal S. R. Patel V. K. Singh J. S. (2019). Plant growth promoting *Curtobacterium albidum* strain SRV4: an agriculturally important microbe to alleviate salinity stress in paddy plants. *Ecol. Indic.* 105 553–562. 10.1016/j.ecolind.2018.05.014

[B115] Vurukonda S. S. K. P. Vardharajula S. Shrivastava M. SkZ A. (2016). Enhancement of drought stress tolerance in crops by plant growth promoting rhizobacteria. *Microbiol. Res.* 184 13–24. 10.1016/j.micres.2015.12.003 26856449

[B116] Wang D. Mookherjee M. Xu Y. Karato S.-i. (2006). The effect of water on the electrical conductivity of olivine. *Nature* 443, 977–980. 10.1038/nature05256 17066032

[B117] Wang W. Wu Z. He Y. Huang Y. Li X. Ye B. C. (2018). Plant growth promotion and alleviation of salinity stress in *Capsicum annuum* L. by Bacillus isolated from saline soil in Xinjiang. *Ecotox. Environ. Safe* 164 520–529. 10.1016/j.ecoenv.2018.08.070 30149350

[B118] Wang X. Chen X. Liu Y. Gao H. Wang Z. Sun G. (2011). *CkDREB* gene in *Caragana korshinskii* is involved in the regulation of stress response to multiple abiotic stresses as an AP2/EREBP transcription factor. *Mol. Biol. Rep.* 38, 2801–2811. 10.1007/s11033-010-0425-3 21127996

[B119] Wang Y. Gao C. Liang Y. Wang C. Yang C. Liu G. (2010). A novel bZIP gene from *Tamarix hispida* mediates physiological responses to salt stress in tobacco plants. *J. Plant Physiol.* 167 222–230. 10.1016/j.jplph.2009.09.008 19853962

[B120] Warrence N. J. Bauder J. W. Pearson K. E. (2002). *Basics of salinity and sodicity effects on soil physical properties. Departement of Land Resources and Environmental Sciences.* Bozeman, MT: Montana State University, 129.

[B121] World Bank (2019). *World Population Prospects 2019: Highlights.* Available online at: https://data.worldbank.org/indicator/AG.LND.AGRI.ZS (accessed August 24, 2019).

[B122] Xu Z. Shao T. Lv Z. Yue Y. Liu A. Long X. (2020). The mechanisms of improving coastal saline soils by planting rice. *Sci. Total. Environ.* 703:135529. 10.1016/j.scitotenv.2019.135529 31759722

[B123] Zerrouk I. Z. Benchabane M. Khelifi L. Yokawa K. Ludwig-Muller J. Baluska F. (2016). A *Pseudomonas* strain isolated from date-palm rhizospheres improves root growth and promotes root formation in maize exposed to salt and aluminum stress. *J. Plant Physiol.* 191 111–119. 10.1016/j.jplph.2015.12.009 26759938

[B124] Zhang Z. J. Chen S. H. Wang S. M. Luo H. Y. (2011). Characterization of extracellular polymeric substances from biofilm in the process of starting-up a partial nitrification process under salt stress. *Appl. Microbiol. Biotechnol.* 89 1563–1571. 10.1007/s00253-010-2947-y 21052992

[B125] Zheng D. Chang Q. Li Z. Gao M. She Z. Wang X. (2016). Performance and microbial community of a sequencing batch biofilm reactor treating synthetic mariculture wastewater under long-term exposure to norfloxacin. *Bioresour. Technol.* 222 139–147. 10.1016/j.biortech.2016.09.114 27716565

[B126] Zhou N. Zhao S. Tian C. Y. (2017). Effect of halotolerant rhizobacteria isolated from halophytes on the growth of sugar beet (*Beta vulgaris* L.) under salt stress. *FEMS Microbiol. Lett.* 364:fnx091 10.1093/femsle/fnx091 28460054

